# Detecting gene-gene interactions using a permutation-based random forest method

**DOI:** 10.1186/s13040-016-0093-5

**Published:** 2016-04-06

**Authors:** Jing Li, James D. Malley, Angeline S. Andrew, Margaret R. Karagas, Jason H. Moore

**Affiliations:** Department of Genetics, Geisel School of Medicine, Dartmouth College, Hanover, NH USA; Division of Computational Bioscience, Center for Information Technology, National Institutes of Health, Bethesda, MD USA; Department of Epidemiology, Geisel School of Medicine, Dartmouth College, Hanover, NH USA; Institute for Biomedical Informatics, University of Pennsylvania, Pennsylvania, PA USA; Department of Biostatistics and Epidemiology, The Perelman School of Medicine, University of Pennsylvania, Pennsylvania, PA USA

**Keywords:** Random forest, GWAS, Machine learning, Scale invariant

## Abstract

**Background:**

Identifying gene-gene interactions is essential to understand disease susceptibility and to detect genetic architectures underlying complex diseases. Here, we aimed at developing a permutation-based methodology relying on a machine learning method, random forest (RF), to detect gene-gene interactions. Our approach called permuted random forest (pRF) which identified the top interacting single nucleotide polymorphism (SNP) pairs by estimating how much the power of a random forest classification model is influenced by removing pairwise interactions.

**Results:**

We systematically tested our approach on a simulation study with datasets possessing various genetic constraints including heritability, number of SNPs, sample size, etc. Our methodology showed high success rates for detecting the interaction SNP pair. We also applied our approach to two bladder cancer datasets, which showed consistent results with well-studied methodologies, such as multifactor dimensionality reduction (MDR) and statistical epistasis network (SEN). Furthermore, we built permuted random forest networks (PRFN), in which we used nodes to represent SNPs and edges to indicate interactions.

**Conclusions:**

We successfully developed a scale-invariant methodology to detect pure gene-gene interactions based on permutation strategies and the machine learning method random forest. This methodology showed great potential to be used for detecting gene-gene interactions to study underlying genetic architectures in a scale-free way, which could be benefit to uncover the complex disease mechanisms.

## Background

Genome-wide association studies (GWASs) have revolutionized the strategy for identification effects of single nucleotide polymorphisms (SNPs) on disease susceptibility and detecting genetic architectures underlying complex diseases from large-scale genotyping data, such as type II diabetes, obesity and cancer [1–5]. GWASs have uncovered a great number of disease susceptibility loci, yet we still have very limited knowledge of the genetic architecture of some diseases and therefore cannot accurately predict the disease risk from genetic information [6]. This is challenging due to the consequences of genetic heterogeneity, epistasis (gene-gene interactions) and gene-environment interactions. Traditional methods that have been used to analyze the genetic-disease associations include linear regression, logistic regression, chi-square test, etc. However, these approaches map single loci one at a time to detect main effects, but ignore interactions between genes and environment factors when mapping the relationship between genotypes and phenotypes [4, 6, 7].

As an alternative to commonly used linear models and other classical methods as above, we applied data mining and machine learning methods, such as multifactor dimensionality reduction (MDR), artificial neural network (ANN) and statistical epistasis network (SEN), etc., to detect interactions between different genes, and between genes and environmental exposures during modeling. The concept here is that these methods perform better to capture the non-linear mapping from genotypes to phenotypes [4, 8–11]. Multi-locus analysis methods, however, can sometimes be computationally challenging when examining all pairwise combinations of SNPs [7]. It can get even more computationally challenging when trying to detect three-way or four-way interactions [7]. Different strategies have been designed to solve such problems, which include applying filter algorithms to reduce the number of SNPs in the analysis by removing redundant SNPs based on the needs, such as Spatially Uniform ReliefF (SURF), and doing pathway analysis to subset the SNP dataset based on similar biological functions [12, 13].

One class of widely used algorithms in machine learning are tree-based methods, such as decision trees (DTs) and random forests (RFs), which belong to the supervised machine learning methods that are used for variable selection, classification and outcome prediction [14]. A single DT grows according to a best binary splitting rule which splits data into two subgroups at each node [15]. For GWAS studies, DT is generated by selecting the best SNP predictor as the node where it best separates samples into two groups; the selection occurs at each further node until, in the default mode, the DT is grown to purity (fully separation of the two classes at the terminal nodes), or until a small number of samples are left at the terminal nodes, to avoid over-fitting [15]. However, purity default method is itself known to overfit. Once the DT is fully learned using training data, the testing data is then applied to the DT by dropping prediction variable values (SNP genotypes) down the tree. DT could output either the predicted class label of the sample based on the most frequent class DT predicts, or quantitatively predicts the mean of the outcomes using regression DTs [14]. In regression mode, the DT uses a local average of the outcome values in each terminal node. RF extends the idea of DTs, a nonparametric tree-based method that uses bootstrap sampling to build an ensemble of DT classifiers and predicts the outcome by aggregate voting from all DTs [16, 17]. Usually, the number of trees and how many splitting rule would apply at each node are used to tune the RF [14].

RF can capture interactions between SNP predictors based on DT modeling on non-linear associations. RF also yields variable importance measures (VIMs) that can be used to rank SNPs as a screening and filtering method. Using RF for studying gene-gene and gene-environmental interactions considering both marginal and interaction effects is appealing [16]. RF was found to be an successful screening tool which outperformed traditional methods such as Fisher’s exact test for detecting risk associated SNPs by using VIMs when interactions exist [18]. People have successfully developed tree-based methods to infer gene regulatory networks [19]. Based on RF, Random Forest Fishing (RFF) has been designed to effectively identify risk factors when considering both marginal effects and interactions using GWAS data [20]. A software package named Random Jungle (RJ) was also designed typically for large-scale association studies which is a fast-implementation of RF and Cordell has applied real data analysis using this package to identify gene-gene interactions [21, 22]. Other than that, Jiang et al. developed a random forest approach, sliding window sequential forward feature selection (SWSFS) algorithm, to detect epistatic interactions in case-control studies according to gini importance [23].

Although RF implicitly considers interactions, further work is required to separate main effects from interactions in RF since VIMs as estimated in RF reflect both main and interaction effects [24, 25]. Unfortunately, previous work has shown that RF is not designed to explicitly test for SNP interactions with hypothesis tests in large genetic datasets, due to the decreasing probability of the co-ocurrence of SNPs predictors in each tree as the feature space is expanded [25]. Therefore, modeling needs to be done carefully to detect interactions and new methodologies need to be designed to capture the pure interactions without main effects between SNPs when modeling with RF.

In this study, we proposed an approach called permuted random forest (pRF) to detect pure interactions between SNPs, which included four steps: training, permutation, testing and ranking. Random forest was trained using original dataset and for each pairwise of SNPs, dataset was later permuted using two methods: one permutation method kept the main effects of the chosen pair of SNPs, the other method kept the main effects and interaction of the chosen pair of SNPs. The subtraction of the two schemes was defined here as the interaction signal between the chosen SNP pair, and therefore measures how different RF outcomes were due to how much the interaction signal contributes to the prediction models. We hypothesized that if two SNPs are highly interacting with each other, the success rate for RF to classify the samples correctly would be affected greatly when removing the interaction between the two SNPs. The stronger interactive SNPs could then be identified by ranking the different classification errors from the above two permutation schemes. We tested our hypothesis systematically on simulated datasets obtained from Genetic Architecture Model Emulator for Testing and Evaluating Software (GAMETES) with different genetic constraints including heritability, number of SNPs, sample size, etc, and achieved good success rates on detecting the interacting SNP pairs even under very low heritabilities. We also applied our approach on two real bladder cancer datasets: one was a 7-SNP dataset with a single pair of SNPs had interaction, the other was a 39-SNP dataset with multiple pairs of SNPs had interaction. We were able to replicate previously identified interacted SNPs by two well-studied methods, MDR and SEN, and also made evidently new discoveries on SNPs with interactions. Finally, we introduced the idea of permuted random forest networks (PRFN), in which we used nodes to represent SNPs and edges to indicate interactions.

## Methods

### Permuted random forest (pRF)

We proposed a method called permuted random forest (pRF) to address these two questions: First, given a SNP dataset, how can we detect the SNP-SNP interactions accurately? Second, how can we analyze all SNPs together into a model to incorporate multi-SNP interactions instead of only analyzing the interactions using the data from the pair of SNPs? In our approach, we quantified the interaction signal by estimating how much the signal contributes to the model prediction power. Our conjecture was that if the interaction between a pair of SNPs contributes greatly to the phenotype prediction, meaning that deleting the interaction causes an increase in prediction error rate, then there was probably a strong interaction between this pair of SNPs. To test if a pair of SNPs have strong interactions, the prediction error rates were obtained from the two testing datasets that were generated from two permutation schemes as shown below. For each pair of SNPs, one testing dataset had its interaction deleted, while the other testing dataset kept the interaction between this pair of SNPs. Thus, the difference of the two testing datasets was purely the interaction, and the different prediction error rate was caused by the existing interaction between this pair of SNPs.

**Fig. 1:**
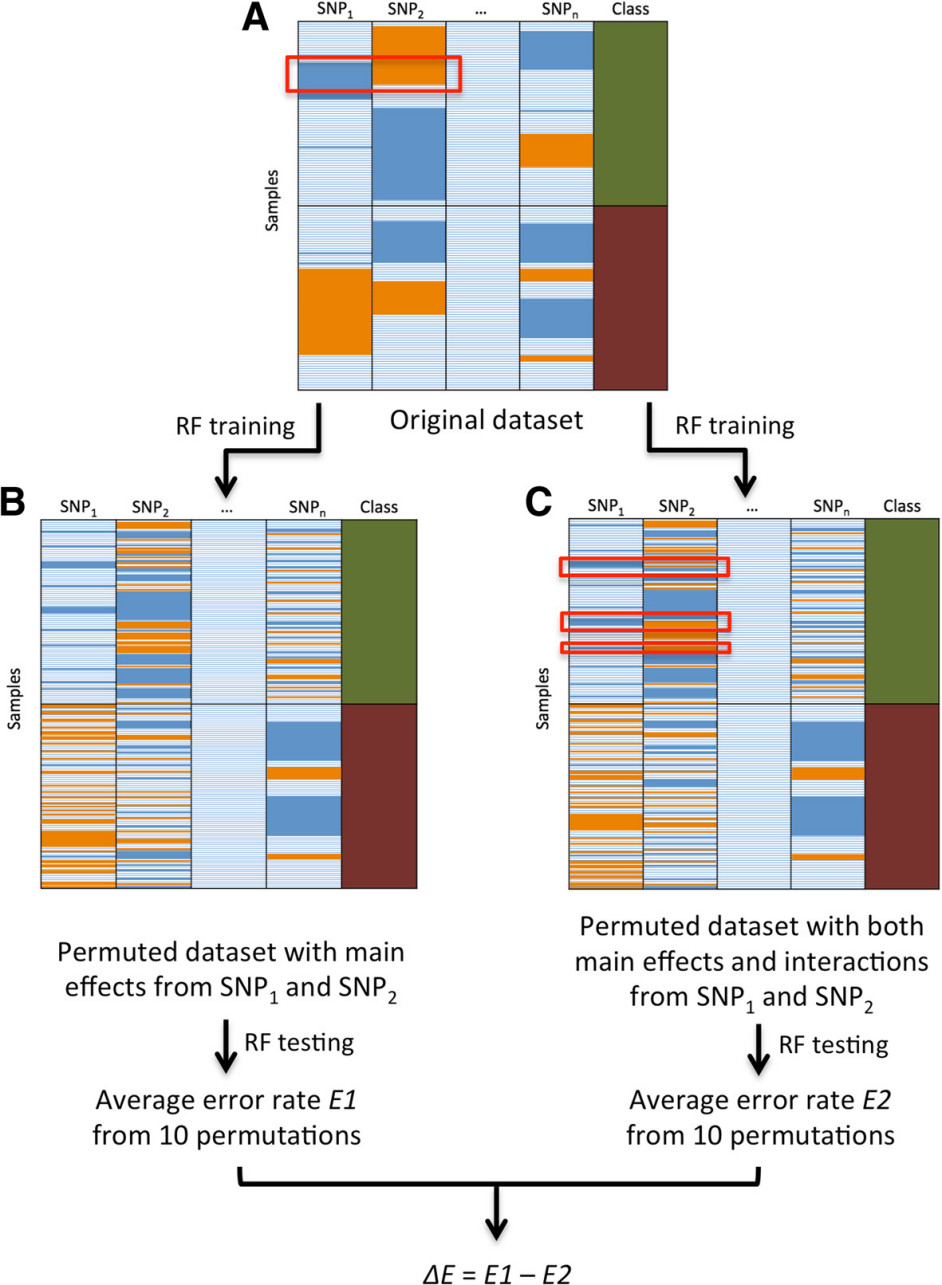
Overview of the permuted Random Forest (pRF). Shown in panel **a** is the original dataset with all the SNP information (0, 1 or 2) and class (cases-control status). Each row represents a sample; different three colors in the SNP columns indicate different genotypes, and two colors in the class column indicate case-control status. **b** shows the first permutation framework that keeps SNPs’ main effects, in which cases and controls are separated, two selected SNP columns shuffle the information separately within each class. **c** shows the second permutation framework that keeps SNPs’ interaction and main effects, in which cases and controls are separated, two selected SNPs shuffle their information together by keeping their genotype combinations, separately within each class. RF is trained using original dataset and tested using the datasets from the above two permutation schemes. Error rates are calculated by averaging the classification errors across all samples. The same process is repeated 10 times and the error rates are averaged from 10 permutation results. The average classification error from the first permutation framework is named *E1*, while the average classification error from the second permutation framework is named *E2*. The whole process is repeated on all pairs of SNPs and the difference in average error rates (*Δ* *E = E1 - E2*) are calculated and ranked to identify the top candidates

Our approach consisted of four steps of (1) training, (2) permutation, (3) testing and (4) ranking using machine learning algorithm RF. In the first step training, RF was trained using the whole original SNPs dataset. We used *‘randomForestSRC’* package in R with the settings *nsplit = 0*, *ntree = 100* and the rest as default, which was a well-established package for carrying out random forest analysis for survival, regression and classification [26]. The RF structure from the training stage was retained, and the structure was later be used for testing on the permuted datasets. The original dataset was shown in Fig. 1a, each column in the dataset represented a SNP while the last column represented the phenotypes; each row represented a sample.

In the second step of permutation, for each pair of SNPs independently, we carried out two permutation strategies to generate two testing datasets. The difference between the two testing datasets was merely the preservation or deletion of the interaction between the pair of SNPs. Previously, Greene et al. designed an explicit test of epistasis to remove SNP interactions, which was based on a permutation method [27]. In their approach, data rows were sorted by class into cases and controls, permutations were then performed in each column within each class to remove any interactions between SNPs in each class [27]. The independent main effects of SNPs were preserved due to the consistent genotype frequencies within each class before and after the permutation [27]. Our two permutation frameworks were motivated from Greene’s method. In our first permutation strategy, data rows were sorted by class into cases and controls, one pair of SNPs were selected and both of their genotypes (0, 1 or 2) were shuffled within each class. By doing this, the interaction between the two SNPs was removed, but the main effects from the two SNPs were maintained. In our second permutation strategy, the same two SNPs were permuted by maintaining their interactions and main effects. In more detail, data rows were sorted by class into cases and controls, and their genotypes (0, 1 or 2) were shuffled together by keeping the combination of SNP information within each class. As shown in Fig. 1a and c, the interaction pattern between SNP _1_ and SNP _2_, indicated inside red rectangles by the blue-orange pattern, was consistent before and after our second permutation strategy. For each pair of SNPs, the above two permutation frames were repeatedly applied to generate two testing datasets. It is also worth mentioning that the interactions among other non-selected SNPs were preserved in both of the permutation frameworks since all the SNPs were considered for the model. This is an advantage of our method since other interactions may have direct or indirect effects on the selected SNPs.

In the third step of testing, for each pair of SNPs independently, the prediction error rates from the two testing datasets were calculated using the method below. For each pair of SNPs, both of the permuted datasets (contained both of the permuted pair of SNPs and the rest of the non-permuted SNPs) were tested using the retained RF structure (from training) to detect classification errors. RF determined class membership of each sample by majority voting from all trees. Classification was correct if the voted class was the same as the original dataset. Classification error was calculated by averaging the classification error from all samples. Permutation was repeated 10 times and the average classification error was calculated from all permutations. We named the average classification error from the first permutation framework, *E1*, in which the testing dataset did not maintain the interaction between the two selected SNPs. We named the average classification error from the second permutation framework, *E2*, in which two selected SNPs maintained both main effects and interaction. Therefore, the subtraction would be the interaction signal that left between the two permutation schemes.

In the last step, after each pair of SNPs independently was permuted using the above two permutation schemes and tested to get the prediction errors, the error rate difference (*ΔE = E1 − E2*) was calculated for each pair of SNPs. The *ΔE* was used to define the strength of the interaction exists, since omitting a strong interaction could have a strong affect on classification power. The larger the *ΔE* was, the stronger interaction signal was indicated for that pair of SNPs. The *ΔE*s were ranked and the pair of SNPs with the largest *ΔE* having the strongest interaction among all SNPs, or we could identify the top interactive SNPs given a particular threshold. For simulation studies, the same process was repeated on the 100 replicate datasets in order to calculate the overall success rate of interaction detection using our approach.

### Multifactor dimensionality reduction (MDR)

Multifactor dimensionality reduction (MDR) is a very popular method that can accurately identifies gene-gene and gene-environmental interactions, which is nonparametric and model-free [8, 28]. To carry out an MDR analysis, a group of n genetic attributes or environmental factors are first selected from all provided factors as the model. All possible combination of the n factors are then represented in the n-dimensional space. The ratios of cases vs. controls are calculated in each condition of the n-factor combinations. The n-dimensional space could be reduced to one dimensional following the grouping rule: the spaces that have the number of cases more than controls are classified as higher risk group, while the spaces that have the numbers of controls more than cases are classified as lower risk group [8, 28]. The classification method can then be used on on this single variable at the reduced dimension space. Traditionally, MDR uses 10-fold cross validation. For each cross validation training set, models are ranked using balanced accuracy. The number one ranked model is the winner for this round. After the 10 rounds, the model with the plurality of wins across the training datasets is the overall winner for that model size. MDR has a lot of advantage. It reduces the dimensionality to one thus makes it easier for the later classification. It is non-parametric, where no parameters are estimated, which is a big advantage over lots of traditional parametric statistical methods. It is also model-free, where no genetic model is assumed and thus give it great utilization on studying the disease where no inheritance-model is known for that or those models are very complicated [8, 28]. Over the past decade, a lot of method and tools has been contributing to MDR to make it use more widely, such as a lot of filter approach to MDR, and a lot of wrapper approaches [29].

### Statistical epistasis network (SEN)

Statistical Epistasis Network (SEN) uses network science to globally study the interactive gene-gene interactions exist in the large GWAS dataset [10]. Each node in the network represents a SNP, and each edge represents the SNP-SNP interaction if the level of the interaction between the two SNPs passes the threshold. The network is mathematically formalized based on the information gain theory [10]. The authors of the SEN detected a 39-SNP connected components as the largest interactive SNPs using a bladder cancer set that contains 1500 SNPs [10]. SEN has several achievements. First, this method could bring the analysis into higher level by finding the large connected components in a global scale. Second, this method possess a scale-free topology and could be highly robust since this is a more natural way for the genetic networks [10].

### Genome-scale integrated analysis of gene networks in tissues (GIANT)

Genome-scale Integrated Analysis of gene Networks in Tissues (GIANT) is a user friendly interface which provides the interactive visualization of the tissue specific networks [30]. The genome-wide functions interaction networks were built from a collection of datasets that covers thousands of experimental results that were extracted from more than 14,000 different publications [30]. The 144 tissue or cell lineage specific contexts were selected across the datasets and network-wide association study (NetWAS) was also developed to analyze the functional networks [30]. This could tremendously helpful to study human disease since most of the human diseases are the result of the gene interactions happened within a particular cell lineage or tissue. Carrying out the network in a tissue specific specific way could increase the accuracy of the results.

### Genetic architecture model emulator for testing and evaluating software (GAMETES)

Genetic Architecture Model Emulator for Testing and Evaluating Software (GAMETES) is a user-friendly software designed by Urbanowicz et al. for simulation studies [31]. GAMETES can generate random, pure and strict *n*-locus models. Pure models are defined as *no single locus displays a marginal effect* and strict models refer to *no subset of the n-locus are predictive of phenotype information* [32]. Such a simulation scheme is preferred here, since more traditional methods are computational expensive and, more importantly, are unlikely to yield pure and strict epistasis models, as defined above. Specifically, GAMETES generates models using specified genetic constraints, and these can include the choice of different heritabilities, minor allele frequencies and population prevalence [31]. Data and models constructed this way can also be ranked by the relative ease of detection metric (EDM), a score that is calculated directly from the model itself, and can lead to further generation of models based on the needs of simulation studies [33]. In this regard, it is notable that GAMETES also involves a data simulation strategy which can quickly and easily generate an archive of simulated datasets for each given model, which, in turn is helpful for further simulation studies [31]. In addition, different sample sizes are later selected when models are used to generate an archive of simulated datasets.

### RandomForestSRC

“RandomForestSRC” is a R package developed for doing survival, regression and classification using machine learning method random forest. Survival forests can grown for right-censored survival data, while regression and classification forests can grown based on either categorical or numeric response [26]. Splitting rules could be selected by users as deterministic or random. Variable selection is implemented by minimal depth variable selection [26]. This package also has the function of the imputing missing data, however, to keep the results comparison consistently we did not use this function in our analysis. This package could runs in both serial and parallel modes, which could be chosen by users [26].

### Datasets

#### Simulated study design

In order to systematically evaluate our method for detecting SNP-SNP interactions, simulated datasets were first considered. Such simulated data provides better control and understanding of the interaction detection through a systematic evaluation process. Hence for this purpose, epistatic 2-locus SNP-disease models and an archive of datasets for each given model were generated using the GAMETES. Specifically, GAMETES was used to generate 100,000 random, strict and pure genetic models for each of 8 different combinations of genetic constraints that are differed by number of locus (SNPs) of 2, heritabilities of 0.001, 0.005, 0.01, 0.05, 0.1, 0.2, 0.3 or 0.4, minor allele frequency (MAF) of 0.2 and population prevalence that is allowed to vary. For each of the 8 genetic constraints combinations, 100,000 models were ranked by EDMs and the models with the highest and lowest EDMs were selected as the two models for data simulation [33]. For each selected model, we simulated 100 replicate datasets under the sample size 2,000 or 4,000 with balanced cases and controls and using different total numbers of SNPs 5, 10, 15, 20 and 25. All together, we generated a total of 16,000 (8 (heritabilities) × 2 (EDMs) × 2 (sample sizes) × 5 (number of SNPs) × 100 (replicates)) datasets, which were used for method evaluation. Each dataset contained one pair of highly interacted SNPs, named *M0P0*, *M1P1*, and the rest of SNPs were named *Nx*. We calculated the success detection rate of our method pRF by observing how often the datasets among the 100 datasets lead to detection of the interacting SNP pair, *M0P0* and *M1P1*.

#### 7-SNP bladder cancer dataset

The cases in the dataset were selected from people who were diagnosed with bladder cancer among those between 25–74 years old and from July 1994 through June 1998 in the New Hampshire State Cancer Registry. Controls were chosen from population lists in New Hampshire Department of Transportation (age ≥ 65), population lists in Centers for Medicare & Medicaid Services (CMS) of New Hampshire (age < 65), shared controls from a non-melanoma skin cancer study with diagnostic period of July 1993 to June 1995, and with additional controls assigned to match the cases on age and gender [34]. Informed consent was obtained from all participants. To collect the samples, DNA was isolated using *Qiagen genomic DNA extraction kits* (QIAGEN Inc.) from peripheral circulating blood lymphocyte specimens, and genotyping was performed using SNP mass-tagging system from Qiagen Genomics and PCR-RFLP. Dataset was preprocessed based on the sufficient DNA concentration and successful genotyping, and the missing phenotypes/genotypes were imputed using the corresponded most frequent phenotypes/genotypes. In this study, we selected a subset of 7 SNPs that had been previously identified to include a highly interacted SNP pair from this data. This dataset includes 560 controls and 354 bladder cancer cases after pre-processing. This dataset had one pair of SNPs had interaction.

#### 39-SNP bladder cancer dataset

The cases in the dataset were selected from people at the same age range who were diagnosed with bladder cancer among from the same start time through longer enrollment period until June 2001 in the New Hampshire State Cancer Registry. Controls were chosen the same way, and shared controls from a non-melanoma skin cancer study with longer diagnostic period of July 1993 through June 1995 and June 1997 through March 2000, and with additional controls assigned as above described [10]. DNA was isolated using the same way as above and genotyping was performed using the GoldenGate Assay system by Illuminaś Custom Genetic Analysis service (Illumina, Inc.). Same data pre-precessing were performed as above. A subset of 39 SNPs was pre-selected from this dataset based on a previous SNP-SNP interaction study [10]. The 39 SNPs have previously been identified as the largest connected components with interactions in the statistical epistasis network (SEN) [10]. This dataset includes 791 controls and 491 bladder cancer cases. This dataset had multiple pairs of SNPs had interactions.

## Results

### Evaluation of our method using simulated data

A total of 160 different datasets, each with 100 replicates were simulated using GAMETES based on the different combination of genetic constraints, that included number of interaction locus (2), heritability (0.001, 0.005, 0.01, 0.05, 0.1, 0.2, 0.3 or 0.4), minor allele frequencies (0.2), number of SNPs (5, 10, 15, 20 or 25), sample size (2000 or 4000), extreme EDM (highest or lowest) and population prevalence that is allowed to vary. The datasets were generated from random, pure and strict epistasis models using GAMETES, with one pair of highly interacted SNPs named *M0P0*, *M1P1*, and with the rest of SNPs named *Nx*. The success rates for identifying the interacting SNPs, *M0P0* and *M1P1*, were calculated for each dataset, and averaged from 100 replicates; see Table 1. It was shown that under most of the genetic constraint combinations, our approach achieved great success rates when identifying interacting SNPs. We also observed that our approach performed better when detecting interaction in models with the highest EDM and higher heritability, in datasets that include less numbers of SNPs, or larger sample size. For the datasets with 5 SNPs, the success rates were 100 % for all the datasets with heritability greater or equal to 0.05.

**Table 1:** Success rates for identification of interaction pairs of SNPs from simulation studies

		Interaction SNP Pair Detection Success Rate			
		Sample Size = 2000		Sample Size = 4000	
SNP Numbers	Heritability	Highest EDM	Lowest EDM	Highest EDM	Lowest EDM
5	0.001	52 %	7 %	70 %	16 %
5	0.005	99 %	28 %	100 %	43 %
5	0.01	100 %	34 %	100 %	68 %
5	0.05	100 %	100 %	100 %	100 %
5	0.1	100 %	100 %	100 %	100 %
5	0.2	100 %	100 %	100 %	100 %
5	0.3	100 %	100 %	100 %	100 %
5	0.4	100 %	100 %	100 %	100 %
10	0.001	8 %	3 %	29 %	1 %
10	0.005	80 %	4 %	99 %	13 %
10	0.01	100 %	8 %	100 %	43 %
10	0.05	100 %	98 %	100 %	100 %
10	0.1	100 %	100 %	100 %	100 %
10	0.2	100 %	100 %	100 %	100 %
10	0.3	100 %	100 %	100 %	100 %
10	0.4	100 %	100 %	100 %	100 %
15	0.001	6 %	0 %	8 %	0 %
15	0.005	51 %	2 %	77 %	6 %
15	0.01	93 %	4 %	100 %	12 %
15	0.05	100 %	92 %	100 %	100 %
15	0.1	100 %	100 %	100 %	100 %
15	0.2	100 %	100 %	100 %	100 %
15	0.3	100 %	100 %	100 %	100 %
15	0.4	100 %	100 %	100 %	100 %
20	0.001	0 %	0 %	2 %	1 %
20	0.005	10 %	2 %	39 %	3 %
20	0.01	49 %	0 %	93 %	12 %
20	0.05	100 %	70 %	100 %	98 %
20	0.1	100 %	100 %	100 %	100 %
20	0.2	100 %	100 %	100 %	100 %
20	0.3	100 %	100 %	100 %	100 %
20	0.4	100 %	100 %	100 %	100 %
25	0.001	1 %	0 %	1 %	0 %
25	0.005	2 %	0 %	10 %	1 %
25	0.01	15 %	1 %	44 %	2 %
25	0.05	99 %	30 %	100 %	87 %
25	0.1	100 %	87 %	100 %	100 %
25	0.2	100 %	99 %	100 %	100 %
25	0.3	100 %	100 %	100 %	100 %
25	0.4	100 %	100 %	100 %	100 %

Success rates for identification of interaction pairs of SNPs with different numbers of SNPs, heritabilities, models with highest/lowest EDMs, under the sample sizes of 2000 or 4000 with balanced cases and controls. The success rates were calculated using 100 replicate datasets. The percentage was calculated as the fraction of correctly detection times of the interacting SNP pair, *M0P0* and *M1P1*, in 100 replicate datasets under each genetic constraint combination of number of SNPs (5, 10, 15, 20 or 25), heritability (0.001, 0.005, 0.01, 0.05, 0.1, 0.2, 0.3 or 0.4), extreme EDM (highest or lowest) and sample size (2000 or 4000)

### Evaluation of our method using 7-SNP bladder cancer dataset by comparing with MDR

The subset of bladder cancer dataset contained 7 SNPs, *XRCC3* (rs861539), *APE1* (rs3136820), *XPD_751* (rs13181), *XRCC1_399* (rs25487), *XPD_312* (rs1799793), *XRCC1_194* (rs1799782), *XPC_PAT* (rs2228001). We applied our permuted Random Forest (pRF) on this dataset and successfully identified the SNP pair, *XPD_751* and *XPD_312*, with the highest different error rates using our two permutation schemes. RF was applied on the whole dataset using R package *‘randomForestSRC’* with the default setting except *nsplit = 0*, *ntree = 100*. The same two permutation strategies were used and repeated 10 times to obtain the average error rate difference *ΔE* for each pair of SNP. As shown in Table 2, by removing the interaction between this two SNPs using permutation strategy, the error rate was greatly increased from 33.76–41.00 %. *ΔE* was 7.23 % for this SNP pair, while the rest ranged from −0.84–0.99 %. Different numbers of permutations, tree numbers and splitting rules had been applied in our method; however, the interaction pair could always be identified (data not shown). To compare our method with other method, MDR was also used on the same dataset to identify the top 2-way models, which indicates the highly interacted SNPs. The last column showed how the MDR ranks the top 2-way models based on the likelihood of having interactions among the SNPs. We found for the most interacted pair shown by our approach, MDR was showing the consistent results by ranking it as the most interactive pairs of SNPs as well.

**Table 2:** SNP interactions identified by permuted random forest (pRF)

SNP pairs	E1	E2	*Δ*E	MDR ranking
XPD.751, XPD.312	41.00 %	33.76 %	7.23 %	1
XRCC3, XPD.751	41.87 %	40.89 %	0.99 %	6
APE1, XPD.312	40.99 %	40.01 %	0.97 %	14
XRCC3, XRCC1.399	34.76 %	33.83 %	0.93 %	2
XRCC3, APE1	35.27 %	34.43 %	0.84 %	7
XPD.312, XRCC1.194	40.14 %	39.53 %	0.61 %	19
XPD.751, XRCC1.194	40.84 %	40.32 %	0.53 %	9
XRCC3, XPD.312	42.22 %	41.73 %	0.49 %	11
XPD.751, XRCC1.399	41.74 %	41.30 %	0.44 %	4
XRCC3, XRCC1.194	35.64 %	35.24 %	0.39 %	18
XRCC1.399, XPD.312	40.35 %	40.25 %	0.10 %	13
APE1, XPD.751	41.14 %	41.05 %	0.09 %	15
XRCC1.399, XRCC1.194	33.63 %	33.63 %	0.00 %	3
APE1, XPC.PAT	35.36 %	35.55 %	−0.19 %	17
APE1, XRCC1.194	34.04 %	34.22 %	−0.19 %	21
XRCC1.194, XPC.PAT	33.38 %	33.65 %	−0.27 %	20
XRCC3, XPC.PAT	35.00 %	35.37 %	−0.37 %	10
XRCC1.399, XPC.PAT	33.01 %	33.43 %	−0.42 %	12
XPD.312, XPC.PAT	40.48 %	40.95 %	−0.47 %	5
XPD.751, XPC.PAT	40.25 %	40.78 %	−0.53 %	16
APE1, XRCC1.399	33.86 %	34.71 %	−0.84 %	8

Classification error rates from datasets obtained by two permutation schemes, *E1* (with main effects only) and *E2* (with main effects and interaction), are shown in the table. Error rate differences were calculated and listed as *Δ* *E*. SNP pairs were ranked by their error rates differences in percentage, indicating the strength of interactions. *SNP pairs* column shows the permuted SNP names. *MDR ranking* column shows the ranking of top 2-way models MDR identified according to the results from our approach

### Evaluation of our method using 39-SNP bladder cancer dataset by comparing with SEN

**Fig. 2:**
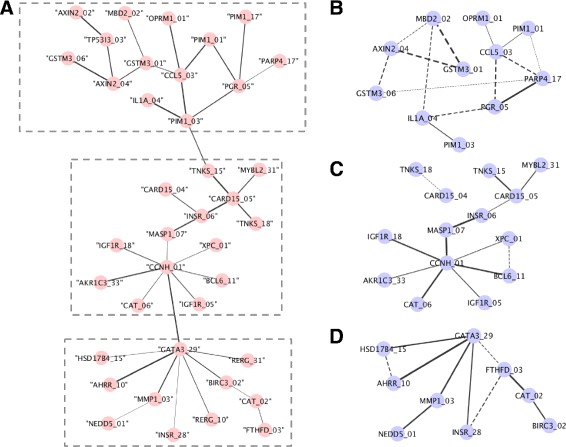
Statistical epistasis network (SEN) and permuted random forest networks (PRFN). **a** shows the largest connected components from statistical epistasis network, which includes 39 SNPs. The largest connected components were divided into three clusters. Permuted Random Forest (pRF) was applied using the SNPs within each of the three clusters separately. **b**, **c** and **d** show the PRFNs built from each cluster. The width of the edges are in proportion to how strong the interactions exist, which are represented by the differences in error rates using our method. The cut-off for the SEN was based on entropy value of 0.013. PRFNs were built using same numbers of edges as in each cluster in SEN

Previously, Hu et al designed a new methodology to detect SNP-SNP interactions using statistical epistasis network (SEN), in which pairwise interactions were calculated based on information theory [10]. In their work, they applied the method to a bladder cancer dataset that includes 1,422 SNPs across 491 cases and 791 controls and successfully detected the largest 39-SNP connected components in which the SNPs were strongly interacting with each other. The 39-SNP pairwise interaction entropy values were obtained from Hu et al and the connected components were rebuilt in *Cytoscape* as shown in Fig. 2a. In SEN, each node represents a SNP in the 39-SNP connected components, while each edge represents an existing interaction with interaction entropy higher than 0.013. From our simulation study, we observed pRF performed better on smaller datasets, therefore, we divided the largest connected components into three clusters based on its structure in Fig. 2a (indicated by grey dotted rectangles). SNP information from each of the three clusters was obtained from the original bladder cancer data with 491 cases and 791 controls. pRF was applied within each of the cluster using R package *‘ramdomForestSRC’* with the default setting except *nsplit = 0*, *ntree = 100* using the same two permutation strategies as previously mentioned. Permutations were done 10 times to obtain the average error rate difference *ΔE*. The *ΔE*s were ranked from largest to smallest and the top candidates were selected as the most interactive SNPs. In order to achieve a fair comparison with previous results obtained by SEN, we used the same number of the edges in each cluster from the original 39-SNP connected components identified by SEN in Fig. 2a as the cut-off numbers of edges in Fig. 2b–d. We was not able to use the same cut-off exact edge value as SEN since the quantification of the interactions is using on a different scale on our method. In our method, each edge represented the error rate difference value *ΔE*. The SNP pairs with the higher difference indicated a strong interaction from the two permutation schemes shown in Fig. 2b–d.

**Fig. 3:**
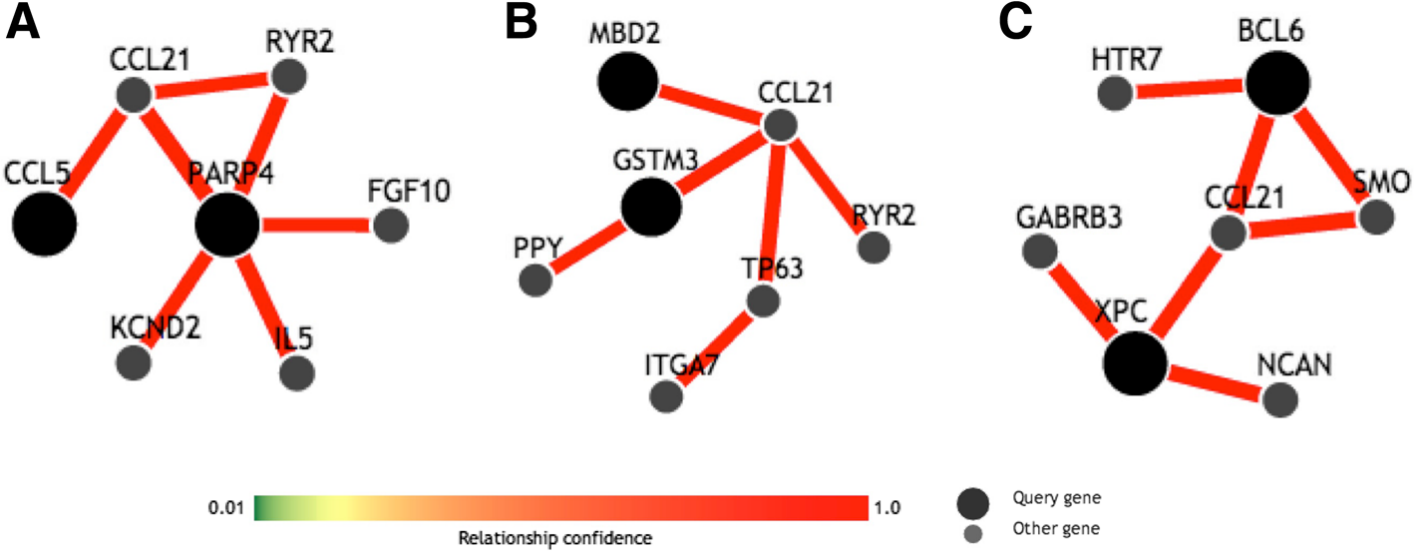
Characterization of newly identified interacted SNP pairs using *GIANT*. Network filters were set as *minimum relationship confidence* 0.8 and *maximum number of genes* 5. Interactions between genes *CCL5* and *PARP4*, *MBD2* and *GSTM*, *BCL6* and *XPC* were characterized using GIANT and the results were shown in panel (**a**, **b** and **c**). **a** shows the network of CCL5 and PARP4; **b** shows MBD2 and GSTM3; **c** shows BCL6 and XPC

We further characterized the newly identified interactions using Genome-scale Integrated Analysis of gene Networks in Tissues (GIANT). As shown in Fig. 3, interactions between genes *CCL5* and *PARP4* in panel A, *MBD2* and *GSTM* in panel B, *BCL6* and *XPC* in panel C were characterized using *GIANT* with network filters set as *minimum relationship confidence* equals to 0.8 and *maximum number of genes* equal to 5. By comparing our method to SEN, we concluded the difference was reasonable.

## Discussion

GWAS provides a powerful approach to discover disease associated genetic variants and a lot of diseases associated SNPs have been discovered via GWAS, yet, that knowledge is still not enough to explain complex diseases 4]. By realizing that most genetic factors function in a complex mechanism when they interact with other genetic and environmental factors, more methods and software packages focusing on detecting the interactions have been used 35]. Early methods to detect interactions include the logistic regression model with interaction terms, joint tests of association and exhaustive searches, but, with good reason, those methods are usually criticized for their inability to deal with high dimensional data 35]. Machine learning and data-mining methods have been developed lately, that use a space of possible models and avoid exhaustively searching the interactions 35,36]. In our approach, we chose a popular machine learning method, random forest, which naturally considers interactions due to its DT structure. All those advantages make RF a suitable method to use in our strategy. Our rationale is that, if allowing the interaction between a particular SNP pair could increase the power to classify the samples using RF, the more the power increases, the stronger the interaction for that SNP pair. Therefore, we designed two different explicit permutation strategies thus to quantitatively characterize the interactions. In summary, we designed a new methodology based on combining both permutation methods and a machine learning method RF to capture gene-gene interactions.

To discuss our result for the simulation study, we found the success rates of our method to detect highly interacted SNP pairs were decreased as the number of SNPs in the dataset increased. For instance, we observed the success rates to be 70 and 30 % under the lowest EDM model at the number of SNP 20 and 25. However, when the sample size was increased from 2000 to 4000, it compensated for the difficulty of detection using datasets with lowest EDM models or the larger SNP size, which improved the ability to detect the interaction pair of SNPs. This was due to the nature of RF when the feature space gets expanded, and it was more generally of the problem of high dimensional data with sparse signal. Also, because this was a permutation-based method, when as the SNP size increased, it took much longer time to sort through all pairwise combinations. However, using parallel mode and running on clusters could help resolve this issue. On the 7-SNP bladder cancer dataset which we compared our result with MDR, we found the most interacted pair shown by our approach was the same as MDR as shown in the “Results” section. We look further for biological functions, *XPD* was found to possess DNA repair capacity (DRC) and studies have found two XPD polymorphisms, *XPD* Asp312Asn and Lys751Gln, had a modulating effect on DRC and there existed possible association between *XPD* Asp312Asn and Lys751Gln polymorphisms in lung cancer 37,38]. All those biological evidence showed that our results are reasonable. We think that our methodology could correctly detect the pair of SNP that has interactions in a dataset containing one pair of interactive SNPs.

Followed by the analysis, we started to wonder how did our method work on a dataset with multiple interactions. To do that, we obtained a dataset from a 39-SNP bladder cancer dataset that was previously identified using SEN from a 1500-SNP dataset to be the largest cluster which had strong interactive functions. The 39-SNP were highly interactive as a network. To compare our result with the previous result, we found there were 28.57 %, 84.62 %, 70.00 % overlapping interacting SNP pairs detected using our method in three clusters in Fig. 2b–d comparing to SEN. Beside those overlapping interactions, we also identified new SNP-SNP interactions that were relatively strong, which included *MBD2_02* (rs1145315) and *GSTM3_01*, *MBD2_02* and *AXIN2_02*. To further check the biological functions, *MBD2* was methl-CpG-binding domain protein 2, which belonged to methyl-CpG-binding domain (MBD) and had been previously identified possessing the function of activating certain promoters by de-methylation, particularly in cancer 39]. *GSTM3* had been found to play a role in detoxification of carcinogens and modulating cancer susceptibility 40]. Based on those biological facts, we think that those two genes may highly be likely to act together in causing cancer. Besides the overlapping interactions and new discovered interactions, there were also some nodes missing in our network comparing to SEN. We think this may be caused by missing some SNP nodes due to the cut-off we chose, since we were choosing the same number of edges as the cut-off comparing to SEN in Fig. 2a as cut-off. Those SNPs included *PIM1_01* (rs10507), *TPS313_03* (rs2303287) and *AXIN2_02* in the top cluster and *RERG_10* and *RERG_31* in the bottom cluster. To get an idea of false positive detection, we also tested some of the gene pairs that were not identified with strong interactions using our method, such as *CCL5* and *PIM1_03* (rs262933), *XPC_01* (rs2228001) and *MYBL2_31* (rs826950), *BIRC3_02* (rs3758841) and *AHRR_10*. None of them were detected with interactions using the same threshold by GIANT (results not shown). To characterize the newly identified interactions we found using our method on this 39-SNP dataset, we used GIANT to further look into this. It is shown from the current databases that those genes have indirect interactions via only one neighbor. Although from the results of GIANT, we did not observe direct interactions between the genes identified using our approach, it is possible that those genes had interactions yet to be demonstrated. Building PRFN on each of the clusters independently might have led to different results compared to building SEN across the whole dataset to get the result of a cluster of 39 SNPs. It is also worth to mention that RF could be used to impute the missing data, which could be better to use over the traditional method of using the average across the samples. However, we wanted to keep the consistency of the same dataset that previously methods used in order to better compare our results. Thus, we did not use the RF to impute the missing data.

We think our approach has several advantages: (1) It is scale-invariant when detecting the SNP-SNP interactions. Random forest itself is non-parametric and our method does not rely on data belonging to any particular distribution. Most of the biological networks are very complicated and have not been studied well to have its mechanism uncovered, therefor we think using a scale-free model could be more reasonable and accurate than using a pre-scaled model. (2) It is based on the idea of permutations and random forest, which are comprehensible and accessible. Both of the permutation strategies are simple and easy to apply to the datasets, and the R package *‘randomForestSRC’* is well designed with clear instructions. This R package could run in both serial and parallel modes, which greatly increase our efficiency by running the jobs on computational clusters. (3) It captures gene-gene interactions by incorporating all SNP predictors into the model. When RF is grown in the training stage, all SNPs are considered in the RF run and RF itself chooses the best SNP at each node. When testing the classification error, the whole dataset with two permuted SNPs information, was dropped on to the trees. Most other methods consider combinations of SNPs instead of involving all SNPs together in the model, thus our method has the advantage of considering interactions that may exist among all other SNPs. (4) RF naturally captures feature interactions based on the DT structures, thus making it a suitable machine learning tool in our approach. (5) One of the best parts of our method is that it does not need a p-value threshold to detect the interactions. Unlike most genome-wide analyses, our approach does not need to perform multi-testing correction since the candidates are identified by sorting and selecting the top candidates. (6) Our approach is highly extendable. The permutation based strategy is not only suitable for application by RF, but could also be used with other machine learning algorithms, such as artificial neutral networks (ANNs) in the deep learning field, which models high-level abstractions from genetic data by the complex architectures. (7) We also think our approach could used on both the categorical data and continuously data due to the fact that RF could be grown using the categorical and continuously data.

We also think our approach does have some drawbacks. (1) Most machine learning methods, including RF and MDR, are not well suited for unbalanced numbers of cases and controls [28, 41]. Thus, if some datasets have a lot more cases than controls, a method may not be able to detect the interacting SNPs. However, one way to solve this issue might be to pre-balance the data before sending it to the machine and repeat the process multiple times before averaging the result; this will be considered in future work. (2) Furthermore, our method performs better in small datasets than large datasets. This is due to the nature of RF when the results are obtained by a explicit permutation. Which is to say, when the RF feature space is expanded, the amount that each predictive variable affects the classification error is decreased. However, while RF is not as good at detecting interactions when the feature space expanded, some other filter algorithms could be used in advance to filter out less-likely candidates for gene-gene interactions. (3) Our method is based on extensive permutations, which could be computationally expensive when applying such a method on high-dimensional data. Running our scheme on a high performance-computing cluster would save a significant amount of time. However, in order to solve the problem from the methodology itself, and for future work, we may think of using synthetic features and applying such a permutation strategy on pathways instead of on single SNPs. Pathways contain multiple SNPs, which are trained in the random forest model as one feature which is including a set of features. Our method ran slower comparing to MDR, however, our method incorporated all SNPs into the model during all the training and testing stages. Thus, all high-order interactions were not missed using our method which could lead the longer time running since MDR does not incorporate such interactions when detecting two-way gene-gene interactions.

## Conclusion

In conclusion, we presented an approach called permuted random forest (pRF), which identified top interacting SNP pairs by estimating how much power the pairwise interactions influence a random forest classification model. Our approach was based on permutation strategies and relying on machine learning method, random forest. Results were shown that our methodology achieved high success rates for interacting SNP pairs detection on an archive of simulated datasets by GAMETES. Our approach was also applied on two bladder cancer datasets and consistent results with MDR, SEN and logistic regression were seen [10, 28]. Furthermore, we built permuted random forest networks (PRFN), in which SNP interaction relationships were clearly shown. We are confident that our approach will be widely applicable for identification accurate gene-gene interactions using SNPs data.
